# Narcolepsy-like pathologic daytime sleepiness in a patient with obsessive-compulsive disorder and attention deficit hyperactivity disorder: a Case Report

**DOI:** 10.3389/frsle.2026.1846349

**Published:** 2026-07-15

**Authors:** Sandra Hackethal, Marco Pecorini, Francesca Duranti, Mauro Manconi, Silvia Miano

**Affiliations:** 1 Sleep Medicine Unit, Regional Hospital of Lugano, Ente Ospedaliero Cantonale (EOC), Neurocenter of Southern Switzerland, Lugano, Switzerland; 2 Faculty of Biomedical Sciences, Università della Svizzera Italiana, Lugano, Switzerland

**Keywords:** attention deficit hyperactivity disorder, central disorders of hypersomnolence, excessive daytime sleepiness, hypersomnia associated with a mental disorder, MSLT, narcolepsy, obsessive compulsive disorder

## Abstract

Excessive daytime sleepiness (EDS) is a frequent complaint in the general population. Other than being a common symptom associated with various sleep disorders, EDS may be a consequence of chronic sleep deprivation or the primary symptom of central disorders of hypersomnolence (CDH). In addition to narcolepsy type 1 (NT1), the other conditions within the CDH spectrum are less well-defined and share considerable clinical and neurophysiological similarities. Herein, we describe the clinical management of a complex case that highlights several challenges in the diagnostic process of a patient with EDS and a history of obsessive-compulsive disorder (OCD). In the absence of other sleep disorders, secondary structural causes, and orexin deficiency as possible causes for EDS, the patient was initially diagnosed with NT2 based on electrophysiological criteria. However, the clinical course, which showed only a partial response to various stimulant medications for subjective and objective daytime sleepiness, led us to question the diagnosis. A detailed psychiatric and neuropsychological assessment revealed, in addition to the previously identified severe OCD and anxiety, a diagnosis of attention deficit hyperactivity disorder (ADHD), subsequently leading to a revised diagnosis of hypersomnia associated with a psychiatric disorder (HPSY). The literature regarding OCD and sleep disorders remains scarce but the connection between ADHD and hypersomnia, as well as narcolepsy, is well-established. Our case report illustrates that a psychiatric and neuropsychological assessment should be considered mandatory for patients with objective EDS.

## Introduction

Excessive daytime sleepiness (EDS) is a frequent complaint in the general population, with its estimated prevalence ranging from 2.5% to 33%, largely depending on the definition used (Pérez-Carbonell et al., 2022). Apart from being a common symptom associated with various sleep disorders, EDS may be a consequence of chronic sleep deprivation or the primary symptom of central disorders of hypersomnolence (CDH). In the CDH group, the pathophysiology of narcolepsy type 1 (NT1) with autoimmune-mediated loss of orexin-A/hypocretin-1 production is well-established, while the mechanisms of narcolepsy type 2 (NT2) and idiopathic hypersomnia (IH) are less understood (Fronczek et al., 2020).

Moreover, the differential diagnosis between NT2, IH, and hypersomnia associated with a psychiatric disorder (HPSY) may be challenging. EDS and hypersomnia can be linked to a variety of psychiatric disorders, with a reported prevalence of up to 50% in major depressive disorder (MDD) and seasonal affective disorders (Kaplan and Harvey, 2009). According to the International Classification of Sleep Disorders, Third Edition, Text Revised (ICSD-3-TR), unexplained EDS in psychiatric patients is classified as HPSY, and it accounts for approximately 11–19% of cases referred for polysomnographic recording (PSG)/multiple sleep latency test (MSLT) (ICSD 3 TR, 2023). On the other hand, mood disorders are frequently reported comorbidities in CDH, with a reported frequency of up to 56.9% in NT1 and 15 to 25% in NT2 (Daniels et al., 2001; Ruoff et al., 2017; Barateau et al., 2017).

Recent studies have also shown a strong overlap suggesting a possible causal relationship between EDS and attention deficit hyperactivity disorder (ADHD), with 61% of patients with CDH and EDS showing ADHD-like symptoms and, conversely, 38% of ADHD patients reporting EDS (Lopez et al., 2020; Lugo et al., 2020).

Obsessive-compulsive disorder (OCD) is a common neuropsychiatric disorder mainly manifesting in childhood and adolescence, with an estimated prevalence of approximately 2–3% (Angst et al., 2004; Ruscio et al., 2010). Patients describe recurrent, intrusive thoughts (obsessions) and/or repetitive stereotypical behavior (compulsions), resulting in marked personal distress and impaired social and professional functioning (Mathes et al., 2019). In contrast to mood disorders, only a few studies have been published regarding sleep complaints in OCD. However, a recent meta-analysis pointed toward significant sleep architecture changes in OCD compared to healthy controls (Nie et al., 2024).

The clinical case we present here combines the above-mentioned comorbidities and diagnostic challenges in a patient with EDS, a history of OCD, and suspected ADHD.

## Case description

In January 2023, P.G., a 21-year-old man, was referred to our Sleep Unit for EDS. He reported experiencing EDS for almost 5 years with gradual symptom onset and without major inciting factors (no major life events, no illnesses, and no vaccinations). At the time of our examination, the patient reported several sleep attacks during the day, which hindered his ability to keep up with his university classes.

He used to sleep during the day, taking naps between 20 and 60 min, but ultimately stopped because he did not feel better or refreshed afterward. He denied experiencing episodes of sleep paralysis, cataplexy, hypnogogic/hypnopompic hallucinations, or weight gain.

Difficulties with concentration and sustaining focus and attention have been reported since childhood, affecting his cognitive and academic performance.

The Epworth Sleepiness Scale (ESS) score was 15/24 (cut off >10). On weekdays, he usually went to bed approximately 11:30 p.m., with a sleep latency of approximately 15–20 min. His sleep was largely uninterrupted until 07:00 a.m., when his alarm was set. He described having difficulty getting up in the morning with sleep inertia lasting >1 h, a condition he has reported since childhood. On the weekends, he postponed his bedtime to approximately 01:00 a.m. with an alarm set for 09:00 a.m. However, he usually turned off the alarm and slept through until 11:00/12:00. Despite prolonging his nighttime sleep up to 11 h on the weekend, he nonetheless experienced sleep inertia and daytime sleepiness.

The neurological examination was unremarkable, and he showed a normal body mass index (20.48 kg/m^2^). No major internist or neurological illnesses were identified in the family history. His personal medical history revealed past enuresis (present until middle school, now only occasionally) and a diagnosis of severe OCD treated with selective serotonin uptake inhibitors (SSRIs, sertraline, dosage and duration unknown) and psychotherapy sessions in 2018/2019, both ultimately suspended by the patient for ineffectiveness.

### Diagnostic assessment and therapeutic intervention

**Figure 1:**
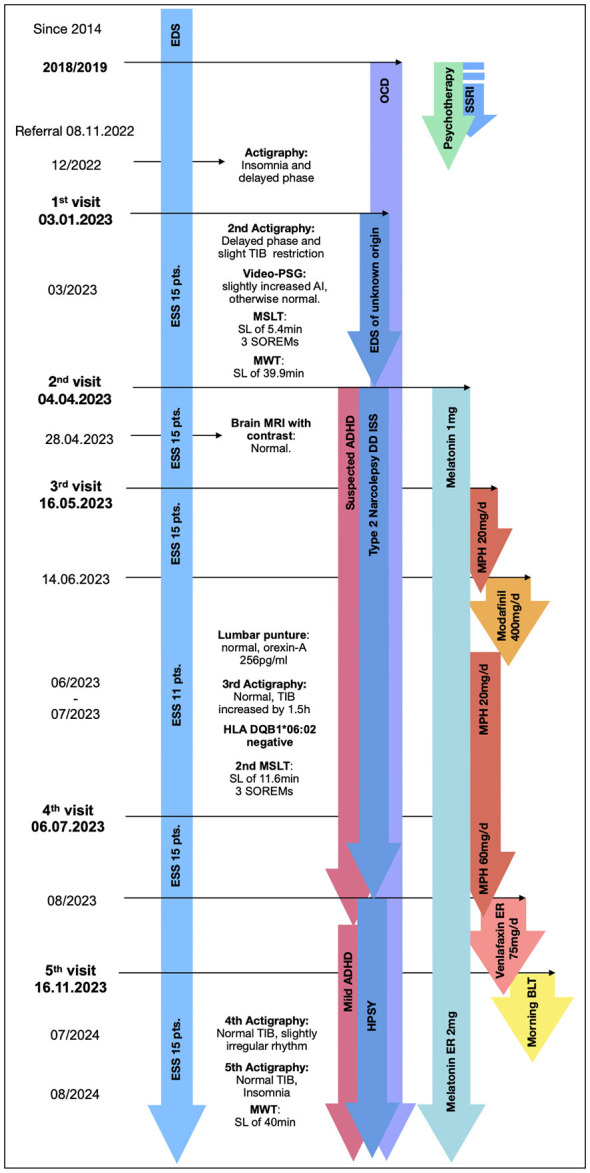
Timeline of the diagnostic and therapeutic process. ADHD, Attention-Deficit/Hyperactivity-Disorder; BLT, Bright-light therapy; EDS, Excessive daytime sleepiness; ER, Prolonged release; HPSY, Hypersomnia associated with a psychiatric disorder; ISS, Insufficient sleep syndrome; MPH, Methylphenidate; MSLT, Multiple sleep latency test; MWT, Maintenance of wakefulness test; OCD, Obsessive-compulsive disorder; pts, points; SL, Sleep latency; SOREMP, Sleep-onset-Rapid-eye-movement sleep; TIB, Time in bed.

For a detailed timeline of the diagnostic assessment and therapeutic intervention, refer Figure 1. A few weeks prior to the first consultation, during the winter holidays, an actigraphy recording (Actiwatch 2 Philips Respironics^®^) was performed, which showed a slight mean bedtime restriction (6 h and 48 min), a delayed sleep-wake rhythm (with bedtimes ranging from 1:00 to 6:00 am), and several brief naps during the day.

**Figure 2:**
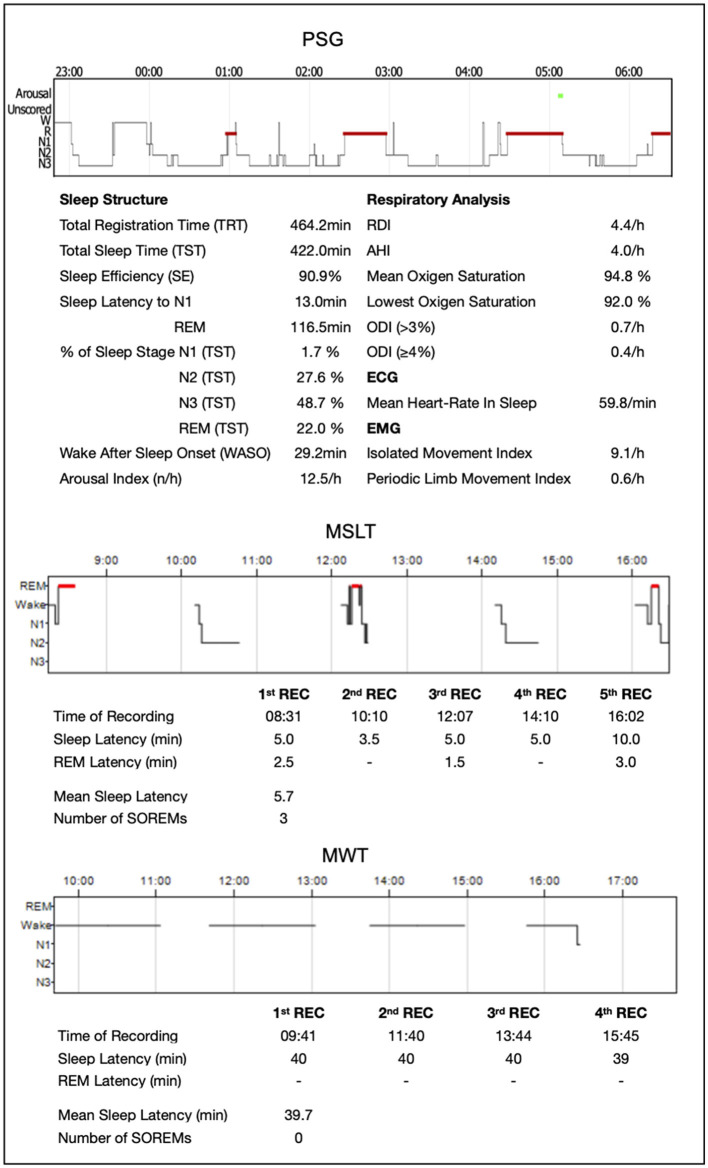
Initial diagnostic assessment (14.03.2022–16.03.2022). In-lab video-PSG assessment (14.03.2022) was followed by Multiple Sleep- latency Test (MSLT, 15.03.2022) and Maintenance of Wakefulness Test (MWT, 16.03.2022) according to our protocol for excessive daytime sleepiness. The patient did not assume any medication in the months leading top to assessment and during testing. AHI, Apnea/Ipopnea-Index; MSLT, Multiple Sleep-latency Test; MWT, Maintenance of Wakefulness Test; ODI, Oxygen desaturation Index; PSG, Polysomnography; RDI, Respiratory Disturbance Index; REM, Rapid-eye-movement; REC, Recording; SOREMs, Sleep-onset REM.

A full sleep assessment consisting of actigraphy, video-polysomnography, MSLT (after the PSG), and maintenance of wakefulness test (MWT) was scheduled in order to better characterize a suspected diagnosis of CDH. Sleep exams were conducted according to the recommended standardized protocols and scoring criteria of the American Academy of Sleep Medicine (AASM 2018a; Berry et al., 2017). The V-PSG showed a largely preserved sleep structure with a high sleep efficiency (90.9%), normal sleep stage distribution, normal arousal index (AI 12.5/h), and normal cardio-respiratory pattern, nocturnal oxygen saturation, and movement pattern (Figure 2). The MSLT, following a total sleep time (TST) of 7 h, showed a mean SL of 5.4 min and 3 episodes of SOREMPs. In contrast, the MWT was completely normal with a mean SL of 39.7 min. Actigraphy recording was comparable to the first registration, with a mean time in bed (TIB) of 6 h and 54 min.

A cerebral MRI resulted in normal findings. CSF hypocretin-1/orexin-A levels, determined in duplicate using the I125 radioimmunoassay kit from Phoenix Pharmaceuticals Inc., were within the normal range (252 pg/ml). The HLA analysis was negative for the DQB1^*^06.02 haplotype.

A provisional diagnosis of NT2 in differential diagnosis with insufficient sleep syndrome was postulated. As the first behavioral intervention, we recommended extending the bedtime to a minimum of 8 h. To facilitate anticipation of his night sleep, we prescribed melatonin 1 mg (at 09:00 p.m.).

**Figure 3:**
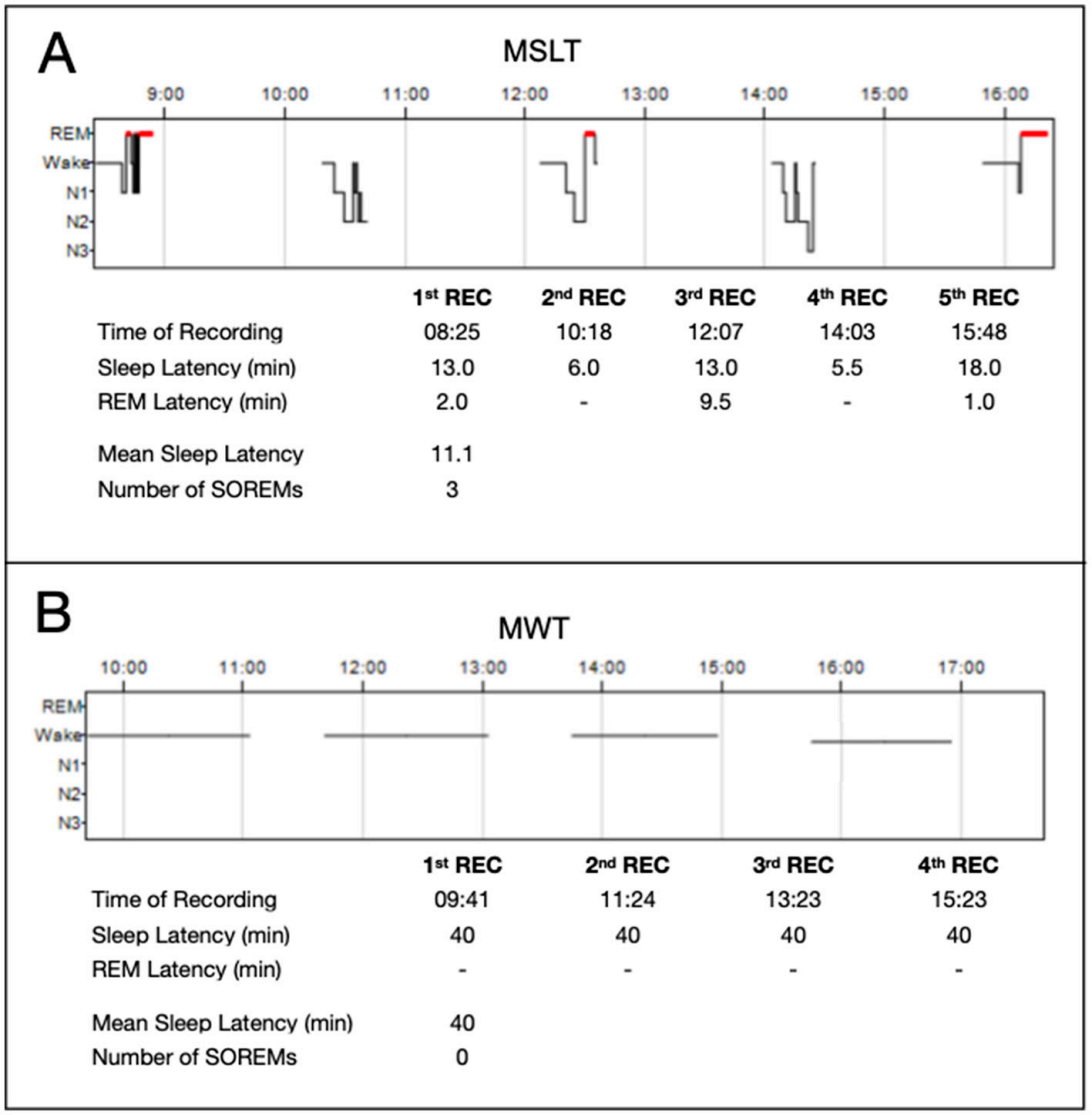
Follow-up assessment. **(A)** After establishing a symptomatic therapy with Ritalin 20 mg/day, the Multiple Sleep-latency Test (MSLT, 03.07.2023) was repeated after 1 week of autography recording with mean time-in-bed of 8 h and 40 min. **(B)** In August 2024, after discontinuation of symptomatic pharmacotherapy for several months for ineffectiveness (see Figure 1 for a detailed time-line), the Maintenance of Wakefulness Test (MWT, 22.08.2024) was repeated after actigraphy recording according of 1 week with mean time-in-bed of 8 h and 30 min. MSLT, Multiple Sleep-latency Test; MWT, Maintenance of Wakefulness Test; REC, Recording; REM, Rapid-eye-movement; SOREMs, Sleep-onset REM.

P.G. was able to increase his TIB to approximately 8–9 h, however, without significant improvement of EDS (ESS 15 pts.), which led us to prescribe a symptomatic therapy with methylphenidate 20 mg/d. The patient reported side effects (tachycardia) and scarce efficacy of the therapy, which was therefore shifted to Modasomil up to 400 mg/d, also ultimately not effective. We again returned to therapy with methylphenidate 20 mg/d. In June 2023, during therapy with methylphenidate 20 mg/d, he repeated an actigraphic recording and MSLT to monitor the therapeutic response. Actigraphy recording showed a mild increase in TIB and TST (8 h and 40 min, and 7 h and 20 min, respectively) and a slight delay in the mean bedtime (00:30 a.m.). The MSLT showed a normalization of mean SL (11 min) with the persistence of three SOREMPs (see Figure 3A).

Based on these findings and the fact that increased TIB and TST did not lead to a convincing, only a transitory clinical improvement in EDS (ESS 11 pts), we settled on a diagnosis of NT2 and further increased the dosage of methylphenidate to 60 mg/d. Again, the patient did not report any benefit from EDS (ESS 15 pts) and suspended the medication after a few months.

Meanwhile, the neuropsychological evaluation did confirm the suspicion of ADHD in a mild form in the prevalent inattentive manifestation. The diagnosis was based on a structured clinical interview and standardized test batteries including the Wechsler Adult Intelligence Scale—Fourth Edition (Wechsler, 2008), *the Barkley Adult ADHD Rating Scale-IV* (“self-report” and “other” based on the patients' mothers' recollection) (Barkley, 2011), the Difficulties in Emotion Regulation Scale (Gratz and Roemer, 2004), the Barratt Impulsiveness Scale (Patton et al., 1995), Mind Wandering (Chiorri, 2019), the Interpersonal Reactivity Index (Maddaluno et al., 2022) and the Test of Attentional Performance—Mobility Version (Zimmermann, 2004).

The patient showed only slight deficits in visuospatial long-term memory with normal results regarding verbal short-term and long-term attentional-executive functioning and memory and visuoconstructive ability, in the greater context of an intellectual profile well above the norm, bordering on giftedness. Unfortunately, the patient did not notice any improvement in memory, concentration, or academic performance with methylphenidate (up to 60 mg/d).

At this point, after multiple ineffective trials with stimulant therapy, all ultimately suspended after a few months of treatment, in a patient with objective EDS but unequivocal neurophysiological findings, we decided to shift our focus to the possibility of EDS in the context of a mood disorder. A psychiatric assessment (Structured Clinical Interview for the DSM (SCID-5) (First, 2015), Beck Depression Inventory—II (Hilsenroth, 2004), and State-Trait Anxiety Inventory—Forma Y (STAI-Y) (Spielberger, 1989), revealed active OCD, anxiety (STAI-Y state 44 /80 pts., trait 50/80 pts., cut-off ≥40) and borderline depressed mood (BDI-II: 12 /63 pts., cut-off ≥13). Venlafaxine 75 mg/d and psychotherapy were prescribed. Based on the clinical course, we ultimately changed the initial diagnosis of NT2 to HPSY. Venlafaxine was discontinued for worsening of the patients' mental state (increase in anxiety and inattention). Considering his intolerance to drugs, a 1-month trial with morning bright light therapy (10,000 Lux 30 min after morning awakening for 30 min) was proposed to the patient to improve his mood and anticipate his delayed sleep-wake rhythm, again with no subjective benefit on mood and EDS.

After 8 months of psychotherapy and without medication, we scheduled a follow-up appointment, including actigraphic recording and MWT. Actigraphy showed moderate sleep maintenance insomnia, while the MWT was normal (see Figure 3B). Upon the patient's request, no further diagnostic assessment was ordered, and no alternative pharmacological strategies were explored.

## Discussion

The present report describes a patient with objective, severe daytime sleepiness, initially diagnosed with NT2 based on international criteria and electrophysiological exams (ICSD 3 TR, 2023). This diagnosis was later changed to HPSY due to the patient's long-standing diagnosis of severe OCD and newly diagnosed comorbid ADHD during follow-up. The ADHD diagnosis provides a better explanation for the EDS.

In our case, the clinical course with only a partial response of subjective and objective daytime sleepiness to various stimulant medications was what made us question the initial diagnosis of NT2.

The existence of NT2 as a separate clinical entity is still a matter of debate. Not only is NT2 a diagnosis of exclusion, but it can also be a transient state, unlike NT1, with patients showing complete remission (up to 45% after 5 years) (Lopez et al., 2017).

A prominent critique raised by experts in the field is that the current diagnostic criteria for CDH show an over-reliance on MSLT criteria, with the MSLT as a technique, moreover, showing various methodological and systematic flaws. Apart from being vulnerable to numerous influencing factors such as age, shift work, sleep deprivation, and medication, the general test–restest reliability for MSLT results varies vastly also between the different CDH. In their 2017 study, Lopez et al. repeated the PSG-MSLT assessment in 22 patients with NT1 and 75 patients with NT2, IH, or undefined EDS after a mean of 1.9 years, with a confirmation of the initial diagnosis in 81.3% of NT1, but only 47.1% in NT2, 25% in IH, and 42.1% in undefined EDS (Lopez et al., 2017). PSG-MSLT results and, therefore, diagnostic classification in the non-cataplexy borderland seem to be unstable and subject to frequent changes, necessitating repeated assessments to provide an accurate diagnosis.

Furthermore, the core electrophysiological EDS criteria of SL < 8 min and ≥2 SOREMPs on the MSLT can not only be found to varying degrees in the single CDH diagnoses but also show substantial overlap with the general population. Approximately 85% of NT1 and (per definition) 100% of NT2 meet the full MSLT criteria, while 60% of HI, 25% of HPSY, and the general population present with SL < 8 min (Plante, 2017; Mignot et al., 2006). Furthermore, some reports suggest that ≥2 SOREMPs on the MSLT can be found in 4–13% of the healthy general population, although these studies being are highly debated (Mignot et al., 2006; Goldbart et al., 2014).

We ultimately did not insist on higher doses or a combination of stimulant therapy in our patients for the side effects experienced already under moderate-dose monotherapy. Most studies investigate the efficacy of stimulant medication in group NT1 and NT2 patients but generally reported mean ESS improvements of 4.7 points (95% CI 1.9–7.4) for modafinil 100–250 mg/day and 5.0 points (95% CI 3.4–6.6) for dexamphetamine 5–60 mg/day (Maski et al., 2021).

In 2018, (Thakrar et al. 2018) published retrospective data on the effectiveness of stimulant therapy (modafinil, methylphenidate, amphetamines, and/or sodium oxybate in mono- or combination therapy) in their CDH patient cohort with *n* = 70 NT1 patients, *n* = 47 NT2 patients, and *n* = 9 IH patients. At last follow-up, after a median of 25.4 months, 39% of patients showed a complete response, 25% a partial response, and 36% a poor response to treatment. An absent treatment response to standard stimulant therapy regimens is therefore not *per se* a reason to question a diagnosis of NT1/NT2/IH, but can point toward an alternative underlying cause.

A detailed psychiatric and neuropsychological assessment of our patient revealed, other than known severe OCD and anxiety, a diagnosis of ADHD. The ICSD3-TR specifically lists mood disorders (including depression, bipolar II disorder and seasonal affective disorder), conversion disorders, schizoaffective disorder, adjustment disorder, and personality disorders as psychiatric conditions for which the diagnosis of HPSY is applicable (ICSD 3 TR, 2023). Neurodevelopmental disorders or anxiety (-related) disorders are not currently listed in the criteria. No electrophysiologic diagnostics or objectification of EDS and/or hypersomnia are required, but HPSY is a diagnosis of exclusion. Electrophysiologic studies are generally expected to be normal, but a meta-analysis found that 25% of patients do in fact present with SL < 8 min (ICSD 3 TR, 2023; Plante, 2017). To date, there are no clear clinical practice guidelines for the treatment of HPSY.

Only a few randomized controlled trials investigating the efficacy of stimulant treatment in HPSY have been published, with the effect of stimulants alone (two RCTs, *n* = 348) or as an add-on to antidepressant treatment (RCT, *n* = 72) either not being significantly different compared to placebo or losing its effect during follow-up (Moderie and Boivin, 2024).

The overall literature regarding a possible association of EDS and anxiety disorders and symptoms is scarcely represented and inhomogeneous (Hayley et al., 2013; Turcio et al., 2022; Wang et al., 2025). For OCD specifically, only a few studies investigating possible sleep complaints have been published, with contradictory reports regarding overall sleep structure, with OCD patients in these studies also suffering from co-morbid depression (Nie et al., 2024; Maddaluno et al., 2022). However, OCD patients without concomitant mood disorders may show SOREMPs in PSG recordings (Kluge et al., 2007; Lange et al., 2012).

In contrast, a strong relationship between EDS and ADHD is well-established, with 61% of patients with CDH and EDS showing ADHD-like symptoms and, conversely, 38% of ADHD patients complaining of EDS (Lopez et al., 2020; Lugo et al., 2020). Since EDS, independent of its origin, has been linked to cognitive dysfunction in general and attention deficits in particular, it is plausible that patients with hypersomnia are prone to show ADHD-like symptoms (Filardi et al., 2021; Hyndych et al., 2025; Kim et al., 2020). A 2020 systematic review found a pooled prevalence of ADHD symptoms in narcolepsy of up to 36.2% in adults and 25% in children, depending on diagnostic criteria, vastly surpassing the rate of 2.5% of adults and approximately 5% of children in the general population. This suggests a close relationship between the two disorders (Kim et al., 2020; Polanczyk et al., 2007; Simon et al., 2009). In a recent study, Ito et al. demonstrated shorter MSLT REM latencies and an overall higher rate of NT2 diagnoses in hypersomnia patients with ADHD (47.8%) as compared to those without ADHD (26.4%). Furthermore, hypersomnia patients with ADHD showed a lower rate of subjective REM-related symptoms despite frequent multiple SOREMPs and did not carry the DQB1^*^06:02 allele (Ito et al., 2018).

The exact pathophysiologic connection between narcolepsy and ADHD remains unclear but dysregulations of the noradrenergic and the hypocretin system have been discussed, with some authors even suggesting hypersomnia with ADHD as a possible subtype of NT2 rather than being a comorbid condition (Kim et al., 2020; Ito et al., 2018). However, since ADHD conceptually is classified as a psychiatric/neurodevelopmental disorder, we would suggest exploring widening the HPSY definition to include a generally more diverse list of psychiatric diagnoses. Based on the current available evidence, the argument for the inclusion of ADHD appears particularly strong.

Our case report ultimately illustrates that in patients with objective EDS, in whom NT1, other neuroinflammatory disorders, and IH have been excluded, a psychiatric and neuropsychological assessment should be mandatory to guarantee a complete assessment and therefore the best possible treatment of affected patients.

### Patient perspective

P.G. was 21 years old when he first embarked on his diagnostic journey with our sleep center, finally looking for a cause and a possible solution to his debilitating, long-lasting daytime sleepiness. He always readily agreed to every step of the diagnostic assessment, even the more invasive procedures such as the lumbar puncture, in the hope of finally receiving an answer and a treatment for his condition. However, with every diagnostic test not resulting in a definitive answer and every unsuccessful therapeutic trial, frustration grew, also aggravated by his uncertainty regarding the final diagnosis. From his point of view, after 2 years of various doctors' visits and diagnostic tests, there was still no real solution for his daytime sleepiness. In the end, upon his request, no further diagnostic assessment was ordered, and no alternative pharmacological strategies were explored. He remains to this day unmedicated, continues external psychotherapy, and tries to adapt and integrate his daytime sleepiness, which still remains unchanged, into his daily life.

### Practice points

1) After an initial comprehensive EDS assessment, repeated re-evaluation and re-assessment of the diagnosis and therapeutic approach might be necessary in clinical practice.

2) In patients with NT2, in whom NT1 and other neuroinflammatory disorders have been excluded, a psychiatric and neuropsychological assessment should be performed, considering the overlaps and comorbidities.

3) A lack of response to standard therapy for NT2 could be a red flag for the presence of other underlying psychiatric comorbidities.

4) We suggest that a broader list of psychiatric including neurodevelopment disorders well established to present with EDS, should be included in the HPSY definition.

## Data Availability

The original contributions presented in the study are included in the article/supplementary material, further inquiries can be directed to the corresponding author.
